# Proteomic analysis of dietary restriction in yeast reveals a role for Hsp26 in replicative lifespan extension

**DOI:** 10.1042/BCJ20210432

**Published:** 2021-12-16

**Authors:** Richard Campion, Leanne Bloxam, Kimberley Burrow, Philip J. Brownridge, Daniel R. Pentland, Patricia Thomas, Campbell W. Gourlay, Claire E. Eyers, Jeff W. Barclay, Alan Morgan

**Affiliations:** 1 Department of Molecular Physiology and Cell Signalling, ISMIB, University of Liverpool, Liverpool, U.K.; 2 Centre for Proteome Research, ISMIB, University of Liverpool, Liverpool, U.K.; 3 Kent Fungal Group, School of Biosciences, University of Kent, Canterbury, U.K.

**Keywords:** aging, mitochondria, molecular chaperones, *Saccharomyces cerevisiae*

## Abstract

Dietary restriction (DR) has been shown to increase lifespan in organisms ranging from yeast to mammals. This suggests that the underlying mechanisms may be evolutionarily conserved. Indeed, upstream signalling pathways, such as TOR, are strongly linked to DR-induced longevity in various organisms. However, the downstream effector proteins that ultimately mediate lifespan extension are less clear. To shed light on this, we used a proteomic approach on budding yeast. Our reasoning was that analysis of proteome-wide changes in response to DR might enable the identification of proteins that mediate its physiological effects, including replicative lifespan extension. Of over 2500 proteins we identified by liquid chromatography–mass spectrometry, 183 were significantly altered in expression by at least 3-fold in response to DR. Most of these proteins were mitochondrial and/or had clear links to respiration and metabolism. Indeed, direct analysis of oxygen consumption confirmed that mitochondrial respiration was increased several-fold in response to DR. In addition, several key proteins involved in mating, including Ste2 and Ste6, were down-regulated by DR. Consistent with this, shmoo formation in response to α-factor pheromone was reduced by DR, thus confirming the inhibitory effect of DR on yeast mating. Finally, we found that Hsp26, a member of the conserved small heat shock protein (sHSP) family, was up-regulated by DR and that overexpression of Hsp26 extended yeast replicative lifespan. As overexpression of sHSPs in *Caenorhabditis elegans* and *Drosophila* has previously been shown to extend lifespan, our data on yeast Hsp26 suggest that sHSPs may be universally conserved effectors of longevity.

## Introduction

Dietary restriction (DR) is the most robust form of environmental manipulation known to increase longevity in a range of organisms [1]. First discovered to extend the lifespan of laboratory rats [2], DR has since been shown to increase the healthy lifespan of many organisms, including yeast, nematodes and flies [3]. DR has been studied extensively in the budding yeast *Saccharomyces cerevisiae* for both chronological and replicative ageing models [4,5]. The most common method of performing DR in yeast is through the reduction in glucose from the standard 2% concentration to either 0.5% or 0.05%, with the latter resulting in the largest replicative replicative lifespan extension [6]. Such studies in budding yeast have led to the identification of conserved genetic pathways linked to DR's longevity and healthspan effects, including Ras/PKA, TOR/Sch9 and sirtuins [1,5,7]. Indeed, direct [8] or indirect [9] activation of the yeast sirtuin, Sir2, by DR-induced changes in NAD/NADH ratios has been proposed as a longevity-promoting mechanism. However, this idea is controversial, as it has been shown that DR does not increase Sir2 silencing activity [10,11] and that DR still increases replicative lifespan in *sir2* deletion mutants [12]. Hence, although significant progress has been made using various model organisms [1], the key downstream effector proteins that ultimately mediate longevity extension by DR remain unclear.

To begin to address this issue, we set out to determine how DR alters cells at the global proteome level using *S. cerevisiae* as a model organism. Yeast are well suited for this, as they are unicellular and hence the confounding issues of tissue-specific gene/protein expression inherent with multicellular organisms are avoided. In addition, the small size of the budding yeast genome (∼12 Mbp) and proteome (∼6000 proteins), coupled with the ease of genetic manipulation facilitates identification and experimental testing of candidate effectors of longevity. Here we describe our use of liquid chromatography–mass spectrometry (LC–MS) to identify differentially expressed proteins under standard (2% glucose) and DR (0.05% glucose) conditions. This confirmed earlier reports that DR causes a major physiological shift towards increased mitochondrial respiration [13], but also revealed that DR inhibits mating by two distinct mechanisms. Importantly, we identify Hsp26 as a protein that is induced by DR and show here that Hsp26 overexpression increases replicative lifespan.

## Materials and methods

### Chemicals and reagents

Materials for yeast culture were obtained from Sigma–Aldrich (Poole, U.K.) and Formedium (Norwich, U.K.). PCR primers were supplied by Sigma Genosys (Havenhill, U.K.), genomic DNA isolation kits were from Invitrogen (Paisley, U.K.); and PCR enzymes/reagents were from Promega (Southampton, U.K.). The custom-made Hsp26 antiserum was made by inoculating rabbits with a synthetic peptide (CVKKIEVSSQESWGN) corresponding to the C-terminal 14 amino acids of the protein preceded by a cysteine for conjugation, and was supplied by Genosphere Biotechnologies (Paris, France). All other materials were obtained from Sigma–Aldrich.

### Yeast strains

BY4741 (*MATa his3Δ1 leu2Δ0 met15 Δ0 ura3Δ0*) was used as the background strain [14]. GFP-labelled strains in this background were used to validate mass spectrometry identifications and were obtained from ThermoFisher (Paisley, U.K.). The isogenic BY4742 strain (*MATα his3Δ1 leu2Δ0 lys2Δ0 ura3Δ0*) was used as a control for shmoo assays. The *HSP26* overexpression plasmid was made by PCR cloning the *HSP26* open reading frame downstream of the *ADH1* promoter in the centromeric p415 vector [15]. BY4741 wild type strains transformed with this plasmid or an empty vector were grown on synthetic dropout media lacking leucine (SD-leu) to select for the *LEU2* marker.

## Proteomics

The sample preparation and proteomic workflows described below were based on our previously published method [16], with modifications. Four independent biological replicate samples for each condition were used for the analysis.

### Yeast culture

Yeast liquid cultures of 10 ml were grown overnight at 30°C in sterile containers. Cultures were then diluted 1/20 in YPD containing either 2% or 0.05% glucose, in a 1 L conical flask and left to grow at 30°C shaking until an OD₆₀₀ of 0.6 (mid-log) had been reached, which took ∼4 h in 2% glucose and ∼6 h in 0.05% glucoseCultures were spun down at 4000***g*** for 5 min and pellets recovered, washed in sterile deionised water, and frozen at −80°C. Yeast pellets were collected until ∼1.5 × 10⁹ cells had been frozen for each condition.

### Sample preparation

Cell samples for a given condition and replicate were recombined into 1 ml bead beating buffer (50 mM ammonium bicarbonate and EDTA-free cOmplete protease inhibitor (Roche)), and centrifuged for 10 min at 4000***g*** at 4°C. The supernatant was removed and 250 µl bead beating buffer added. Cell lysis was achieved by automated glass bead-beating using a MINILYS® homogeniser (Precellys, U.K.), applying 15 × 30 s cycles at 4°C with a 1 min break between each cycle, when lysates were cooled on ice. Lysates were centrifuged for 10 min at 13 000***g*** at 4°C, and supernatant collected. Insoluble material was re-suspended in 250 µl of bead beating buffer, and a small hole pierced in the bottom of the tube using a hot fine needle. Flow-through was then collected via centrifugation for 10 min at 4000***g*** at 4°C and combined with the supernatant. The total volume for the combined fractions was recorded to estimate the number of cells/ml for each condition. A protein assay was then performed to determine the amount of protein within each sample.

### In-solution digest of whole-cell lysates

For protein digestion, a volume equivalent to 25 million cells (∼100–150 µg protein) was diluted to 160 µl with 25 mM ammonium bicarbonate. Proteins were denatured with RapiGest™ detergent (10 µl of 1% (w/v), 80°C for 10 min), reduced using 60 mM dithiothreitol (10 µl, 60°C, 400 rpm shaking for 10 min), cooled on ice and alkylated with 180 mM iodoacetamide (10 µl, incubation at room temperature in the dark for 30 min). Excess iodoacetamide was quenched to prevent over-alkylation by adding excess DTT solution. In-solution digestion was carried out by adding 2 µg of sequencing grade porcine trypsin (Promega, U.K.) per sample, incubating at 37°C overnight with rotation. Trifluoroacetic acid (TFA) was added to the reaction mixture (1% (v/v) final concentration) for 45 min at 37°C to stop the digestion and hydrolyse RapiGest™ detergent. RapiGest™ precipitate and any remaining particulate in the sample was removed by centrifugation at 16 000***g*** for 20 min at 4°C.

### LC–MS analysis

Digests (3 µl) were analysed in a random order using an Ultimate 3000 RSLC™ nano system (Thermo Scientific, Hemel Hempstead) coupled to a QExactive™ HF mass spectrometer (Thermo Scientific). Samples were loaded onto the trapping column (Thermo Scientific, PepMap100, C18, 300 µm × 5 mm), using partial loop injection, for seven minutes at a flow rate of 12 µl/min with 2% ACN 97.9% H₂O, 0.1% TFA. Samples were resolved on the analytical column (Easy-Spray C18 75 µm × 500 mm 2 µm column) using a gradient of 96.2% A (0.1% formic acid) 3.8% B (80% ACN 19.9% H₂O,1% formic acid v/v) to 60% A 40% B over 90 min at a flow rate of 300 nL/min. The instrument was operated in data-dependent acquisition (DDA) mode using a 60 000 resolution full-scan MS scan (AGC set to 3 × 10^6^ ions with a maximum fill time of 100 ms), with the 16 most abundant peaks being selected for MS/MS using a 30 000 resolution scan (AGC set to 1 × 10^5^ ions with a maximum fill time of 45 ms) with an ion selection window of 2 m/z and a normalised collision energy of 30. To avoid repeated selection of peptides for MS/MS, the program used a 20 s dynamic exclusion window.

### Label-free protein quantification

The raw LC–MS files were analysed in Progenesis QI for Proteomics, label-free analysis software which aligns the files and then peak picks for quantification by peptide abundance. The Progenesis QI workflow creates a virtual aggregate run comprising all the data from the individual samples which allows features to be cross identified from other samples, overcoming stochastic sampling limitations of DDA. The software first aligned the LC–MS files and peak picked the aligned peptides. An aggregate file was generated that contained all the peaks from all runs in the experiment so that there are no missing values. Normalisation was performed using the ‘normalise against all proteins' option. The software assumed that most proteins are not changing in abundance and normalisation factors are used to adjust peptide intensities. The peptide list was exported into MASCOT and searched against the UniYeastS288c protein database (with carbamidomethyl cysteine as a fixed modification and methionine oxidation as a variable modification, a precursor mass tolerance of 10 ppm and a product ion tolerance of 0.01 Da) and the peptide lists imported back into Progenesis and assigned to features. Peptide identity matches were at a 1% false discovery rate.

## Bioinformatics

Analysis and visualisation of genetic and protein interactions within the proteomic data was performed with the open-source software Cytoscape (https://cytoscape.org/) and the associated GeneMANIA plugin (https://genemania.org/). GO term statistical enrichment analysis was performed using Panther (http://pantherdb.org/) using a background reference dataset comprising all proteins identified by LC–MS/MS in both 2% and 0.05% glucose conditions. Visualisation of GO term enrichment was performed using GO Slim Mapper (https://www.yeastgenome.org/goSlimMapper).

## High-resolution respirometry

Yeast cell respiration was determined at 30°C using an Oxygraph-2 k system (Oroboros, Innsbruck, Austria) equipped with two chambers. Yeast cells (2 ml) at a concentration of 3.5 × 10^6^/ml, in YP media were added to each chamber and assays conducted in biological triplicate. The chambers were closed and routine respiration was recorded. LEAK respiration was determined by the addition of 150 µM TET, an ATP synthase inhibitor. Uncoupled respiration was then determined by the addition of the ionophore FCCP (12 µM). The addition of 2 µM Antimycin A accounted for non-mitochondrial oxygen consumption. Data were analysed using DatLab software.

## Shmoo assay

Cells were grown in 2% or 0.05% glucose media to an OD_600_ of 0.6 and then 400 µl aliquots were centrifuged for 3 min at 3000***g***. These were then re-suspended in 400 µl of fresh media (2% or 0.05% glucose), spun down and placed in fresh media once more. At this point, 10 µl of 2 mg/ml α-factor or ethanol (vehicle control) was added to the cells, before being placed in an incubator at 30°C. At set intervals, 20 µl aliquots were taken and placed on to a microscope slide and 100 single cells were checked at random to determine the proportion of cells that had started to form a mating shmoo.

## Size-exclusion chromatography

Yeast colonies were grown overnight in 10 ml of YPD broth containing either 2% or 0.05% glucose (w/v). The resulting cells were spun down at 4000***g*** and washed three times with deionised H_2_O. The pellet's wet weight was measured, and aliquots of 30 mg samples were re-suspended in 100 µl of non-denaturing lysis buffer (NDLB) containing protease inhibitor cocktail (Roche) and 4 mM MgATP. The cell suspension was then lysed by bead beating in a Mikro Dismembrator S at 2000 rpm for 5 min. Lysates were cleared by centrifugation at 5000 rpm for 5 min at 4°C, and then at 13 500 rpm for 10 min at 4°C. Size exclusion chromatography was performed on an AKTA purification system (GE Life Sciences) using a HiLoad 16/60 Superdex 200 prep grade column (120 ml). Cleared lysates were diluted to a protein concentration of 1 mg/ml, as determined by Bradford assay, and 400 µl of this was injected into the column. Separated protein samples were collected in 15 ml Falcon tubes in 4 ml fractions for a total of 34 fractions.

## Cell lysate preparation for DR reversal time-course

Yeast colonies were grown overnight in 10 ml of YPD broth containing 0.05% glucose (w/v). These cultures were then diluted in fresh 0.05% glucose media to an OD₆₀₀ of 0.1 and left to grow at 30°C shaking until an OD₆₀₀ of 0.6 had been reached. The resulting culture was washed with water and then split into three aliquots, one of which was processed immediately (0.05% control, time point zero). The other two aliquots were pelleted by centrifugation, diluted into fresh YPD broth containing 2% glucose to an OD₆₀₀ of 0.2 and 0.1 and shaken at 30°C for 3 h and 5 h, respectively, to ensure a final OD₆₀₀ of between 0.5 and 0.7. Samples were processed by resuspending 30 mg of wet weight pellet in 100 µl of hot Laemmli dissociation buffer. Preparation of cleared cell lysates was performed as described above using a Mikro Dismembrator S, after which samples were boiled for 5 min prior to use in SDS–PAGE.

## SDS–PAGE and Western blotting

Proteins were separated on NuPAGE- 4–12% Bis-Tris protein gels (ThermoFisher, U.K.). An amount of 20 µl of each sample was run alongside 10 µl of SeeBlue Plus2 pre-stained protein standard (ThermoFisher, U.K.), at 160 v for 1 h. Gels were stained with SimplyBlue SafeStain (Invitrogen, U.K.). SDS–PAGE gels were transferred to a nitrocellulose membrane (BioTrace, Pall Life Sciences) at 100 V for 1 h in low molecular weight transfer buffer (25 mM Tris, 192 mM glycine, 40% methanol) in a Mini Trans-Blot Electrophoretic Transfer Cell (Bio-Rad, U.K.). Membranes were probed with either custom-made rabbit anti-HSP26 antiserum at 1 : 1000; with mouse anti-GFP antibody (Sigma, U.K.) at 1 : 1000; or with mouse anti-tubulin antibody at 1 : 1000 (Sigma, U.K.). Bands were visualised through enhanced chemiluminescence (ECL) using a Bio-Rad Universal Imager. Densitometry was performed using Image Lab (Bio-Rad, U.K.) and ImageJ.

## Replicative lifespan analysis

This was performed essentially as described previously [17]. Briefly, yeast strains were streaked onto appropriate media and individual virgin cells moved to identifiable grid positions on the agar plate using an MSM micromanipulator (Singer Instruments, Somerset, U.K.). The number of daughter cells produced by each mother cell was then recorded. The plates were incubated at 30°C during working hours, and moved to 4°C overnight. Replicative lifespan was defined as number of daughter cells removed from the mother cell. Statistical analysis of replicative lifespan data was carried out using the online application OASIS 2 [18].

## Results

**Figure 1.**
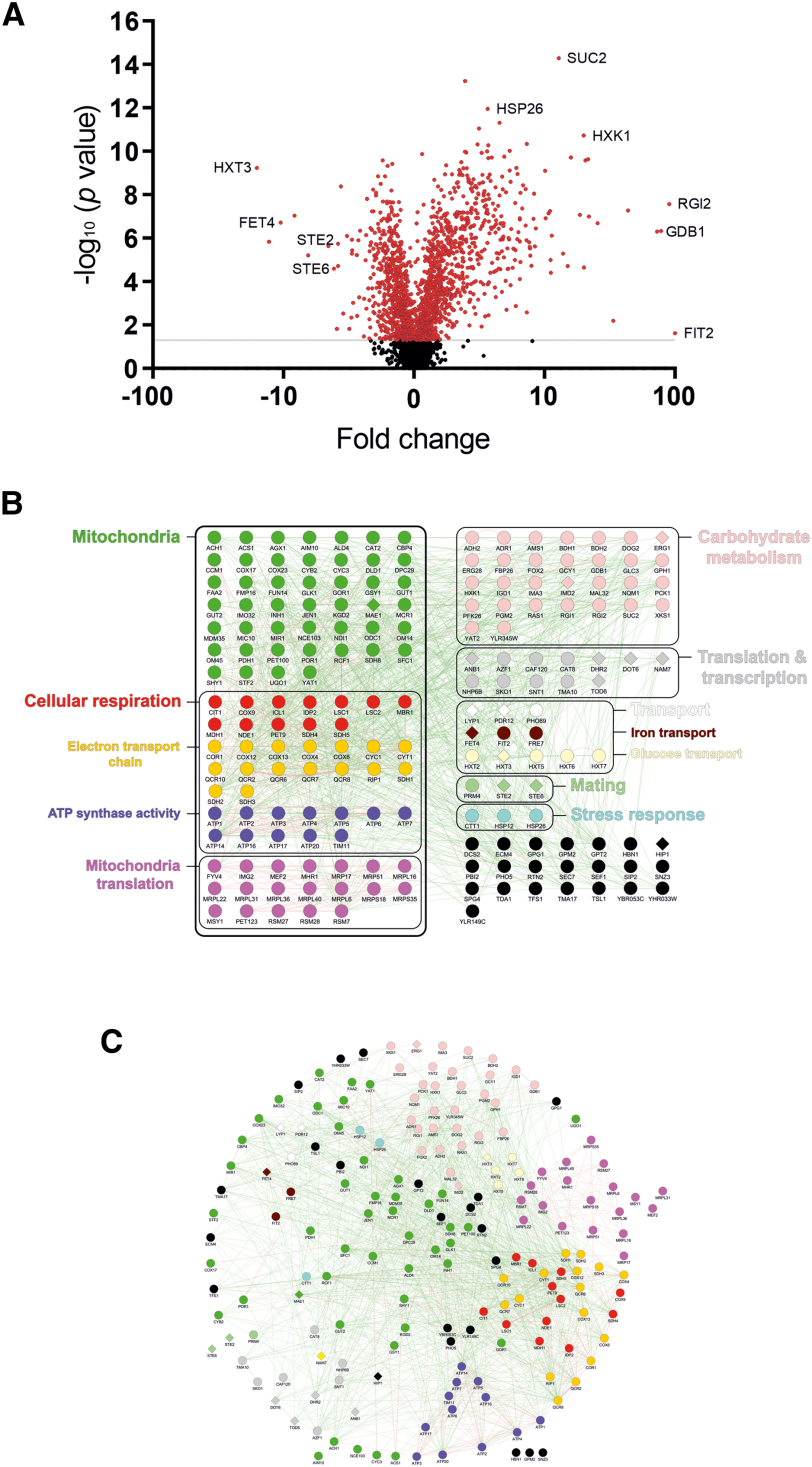
Functional classifications and interactions of DR-regulated proteins. (**A**) Volcano plot showing the distribution of all 2578 proteins identified in our proteomic analysis by fold expression change and statistical significance. (**B**) The 183 proteins with a greater than 3-fold expression change in DR are shown, categorised through their main cellular functions/processes. Circular nodes indicate increased expression in DR, diamond nodes indicate decreased expression during DR. Black circles indicate those proteins that could not be easily grouped with other proteins into functional categories. (**C**) Network diagram showing the genetic (green edges) and physical interactions (red edges) of these 183 proteins. Proteins with similar attributes or similar interactions are shown clustered together in the network.

To identify proteins that are differentially expressed under DR, *S. cerevisiae* BY4741 cells were grown under standard (2% glucose) or DR (0.05% glucose) conditions. Cell lysates were trypsinised and analysed by liquid chromatography–tandem mass spectrometry (LC–MS/MS), identifying 2578 proteins with 2 or more unique peptides (<1% FDR) across the four biological replicates for all conditions (Supplementary Table S1). Of these, 383 proteins exhibited a significant change in expression of at least 2-fold change as determined by label-free quantification, with 183 proteins exhibiting a greater than 3-fold change in levels during DR conditions (Supplementary Table S2). The distribution of all identified proteins by expression change and *P*-value is shown as a volcano plot in Figure 1A.

Focussing on the proteins with at least a 3-fold expression change, 168 were increased and 15 decreased in expression. These 183 proteins can be placed into broad functional categories with many sharing genetic and/or physical interactions with one another (Figure 1B,C; Supplementary Tables S3 and S4). Strikingly, mitochondrial proteins made up a large proportion of this set of differentially expressed proteins. Indeed, analysis of cellular GO terms revealed that most proteins with elevated expression during DR were localised to the mitochondria; and that proteins increased by DR were over-represented in mitochondrial processes, such as cellular respiration (Supplementary Table S3; Supplementary Figure S1).

**Figure 2.**
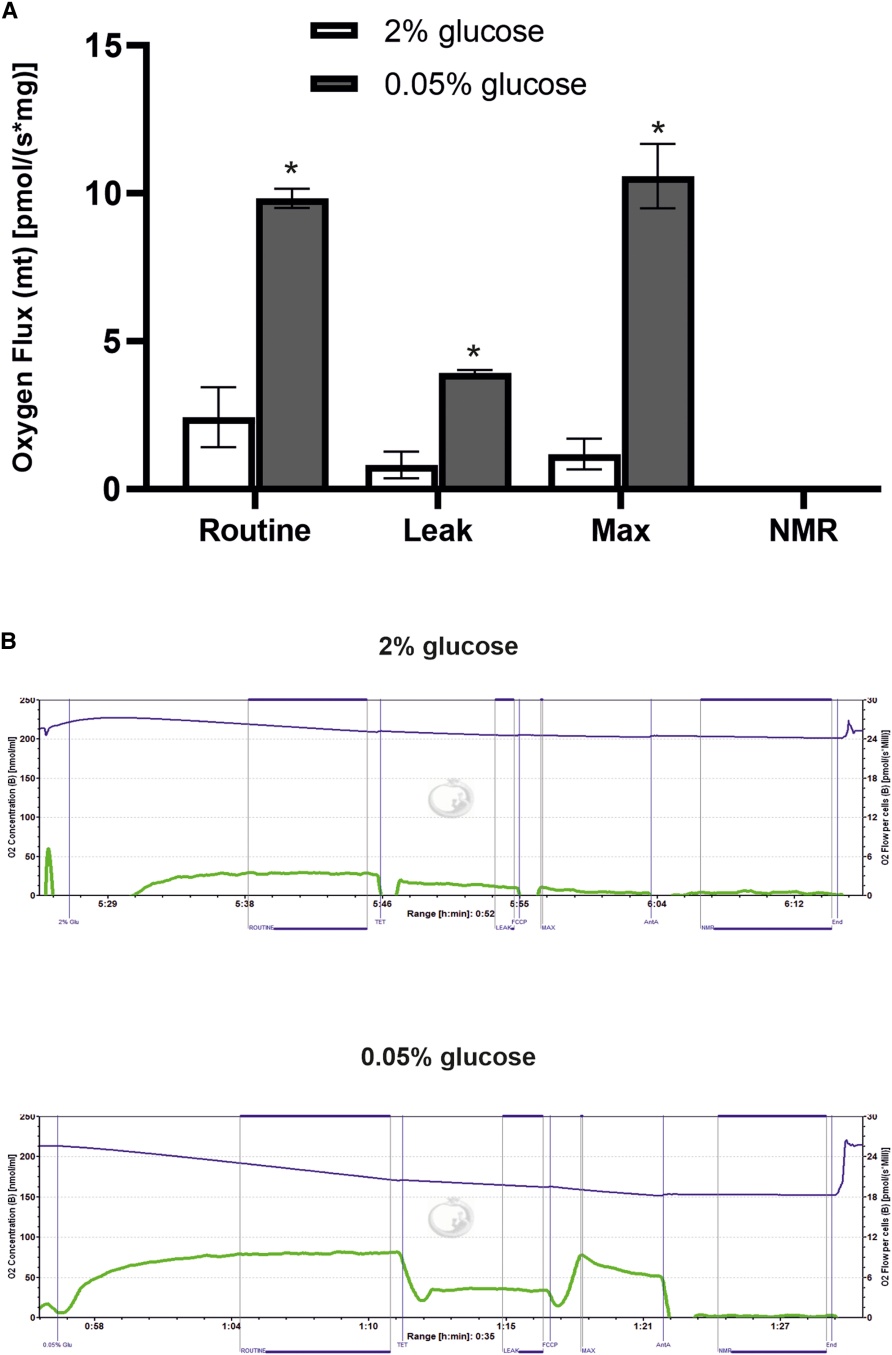
DR increases mitochondrial respiration. BY4741 cells were grown to mid-log phase in either 2% or 0.05% glucose, and injected into sealed chambers of a high-resolution Oxygraph-2k respirometer. Respiration was then measured through oxygen consumption in these chambers. The inhibitors TET, FCCP and antimycin A were injected into each chamber at set intervals to diminish specific mitochondrial functions in order to calculate the cell's routine mitochondrial respiration (standard activity), mitochondrial leak (activity without active ATP synthase), maximal mitochondrial activity (if electrons could free flow through respiration) and non-mitochondrial respiration (NMR). (**A**) Quantification of respiratory activity from three independent experiments demonstrates that all three mitochondrial respiration functions are increased by DR (* *P* < 0.05). (**B**) Example Oxygraph traces from one of the three replicate experiments. The blue line indicates the oxygen consumption, while the green line shows the relative activity of the mitochondria based on the speed of oxygen consumption.

To validate these results functionally, cells were grown under standard and DR conditions and analysed for respiratory capacity using an Oxygraph-2k respirometer. Respiratory function was measured in four categories: routine functional capacity (routine), functional capacity without functioning ATP-synthase (leak), maximum functional capacity (max) and non-mitochondria respiration capacity (NMR). All three mitochondrial functional capacity categories were increased several-fold in DR conditions (Figure 2). Therefore, the DR-induced increase in mitochondrial protein expression evident in our proteomic analysis is mirrored by increased mitochondrial respiratory activity.

**Figure 3.**
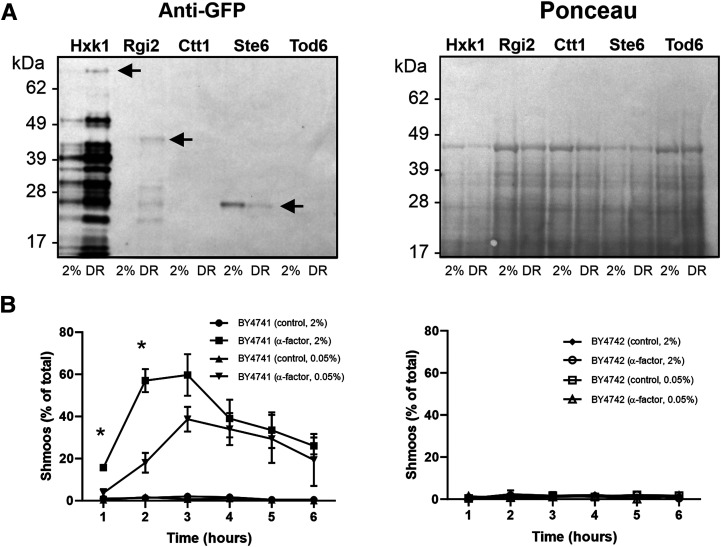
DR inhibits mating by two distinct mechanisms. (**A**) Cells expressing the indicated GFP-tagged proteins as the sole chromosomal copy were grown in 2% or 0.05% glucose. The resulting lysates were then western blotted and probed for GFP (left panel) or stained for total protein with Ponceau S (right panel). Arrows indicate bands corresponding to the predicted size of full-length Hxk1-GFP and Rgi2-GFP, confirming that they are both up-regulated by DR. The arrow for Ste6–GFP indicates free GFP remaining after trafficking to and degradation in the vacuole, confirming that Ste6 is down-regulated by DR. Ctt1–GFP and Tod6–GFP were not detected by the GFP antibody. (**B**) Cells were grown in 2% or 0.05% glucose media to an OD_600_ of 0.6 and then exposed to 50 µg/ml of purified α-factor or ethanol (vehicle control). After each hour, 100 cells from each condition were analysed by microscopy to determine the proportion of cells that had started to form a mating shmoo. Data shown are pooled from three independent experiments (300 cells per datapoint). The left panel shows BY4741 MATa cells, which produced significantly more shmoos in standard conditions than DR conditions at the 1- and 2 h timepoints (* *P* < 0.05). The right panel shows isogenic BY4742 MATα cells, used as a control because they do not express the α-factor receptor, Ste2.

After mitochondrial proteins, the next largest group of proteins whose expression was increased by DR was those involved in carbohydrate metabolism (Figure 1B). To validate our proteomics data, we grew selected GFP-tagged strains under standard and DR conditions and then immunoblotted for GFP (Figure 3A). For Hxk1 (hexokinase), this revealed a DR-induced increase in a band of ∼80 kDa, the predicted size of an Hxk1-GFP fusion protein. Several other lower molecular mass bands detected by the GFP antibody were also increased by DR, and are likely to be proteolytic fragments of the full-length Hxk1-GFP fusion protein. A similar pattern was observed with Rgi2, a small 19 kDa protein involved in energy metabolism under respiratory conditions [19]. Quantitative densitometry of the GFP signal normalised to total protein (as determined by Ponceau S staining, shown in Figure 3A) revealed that the DR-induced increase in expression of both Hxk1-GFP and Rgi2-GFP was ∼5-fold. Although this is less than the fold increases detected by our quantitative LC–MS data (Supplementary Table S2), it nevertheless confirms that DR increases the expression of proteins involved in carbohydrate metabolism.

We also used the GFP-tagging approach to validate proteins that were down-regulated by DR. For some proteins, such as Tod6, we failed to detect a GFP signal, but for Ste6–GFP there was a clear DR-induced reduction in the level of a GFP-immunoreactive band of ∼27 kDa (Figure 3A). As this is the molecular mass of free GFP, it likely reflects the previously reported recycling of Ste6–GFP to the vacuole/lysosome [20,21] and it's consequent proteolysis, as it is well established that GFP is resistant to lysosomal degradation [22]. Ste6 is a MATa-cell-specific plasma membrane transporter that pumps a-factor pheromone out of the cell to signal mating competence to cells of the opposite mating type (MATα). Hence, the reduced expression of Ste6 in DR would be predicted to lead to decreased mating activity, due to the consequent reduction in a-factor secretion.

Intriguingly, other proteins involved in mating were also observed by LC–MS to be down-regulated by DR, including the α-factor pheromone receptor, Ste2. When the MATa-cell-specific Ste2 protein binds to α-factor, this causes a characteristic plasma membrane deformation, known as a shmoo, to form in an attempt to fuse with the cell of the opposite mating type that released the α-factor [23,24]. To test if mating capabilities during DR are decreased due to the down-regulation of Ste2, BY4741 MATa cells were exposed to purified α-factor and the percentage of cells forming shmoos assessed by microscopy. This revealed a significant decrease in the proportion of cells forming shmoos under DR compared with standard conditions (Figure 3B), with a greater than 3-fold reduction in shmoos being observed at 2 h. At later time points, the difference between standard and DR conditions diminished, presumably due to the α-factor being progressively degraded by secreted extracellular Bar1 protease [25]. No shmoo formation in response to α-factor was observed using BY4742 MATα cells under any conditions, demonstrating the specificity for MATa cells (Figure 3B). Taken together, these data suggest that DR reduces mating by two separate mechanisms: decreased a-factor secretion via down-regulation of its transporter, Ste6; and decreased α-factor binding via down-regulation of its receptor, Ste2.

Although most DR-regulated proteins were linked to respiration and metabolism, two heat shock proteins were strongly induced by DR: Hsp12 and Hsp26. Hsp12 is required for replicative lifespan extension by DR in yeast [26], but is not conserved in metazoans. Hsp26, in contrast, is a member of the evolutionarily conserved, α-crystallin domain-containing classical small heat shock protein (sHSP) family [27]. Intriguingly, sHSPs have been shown to increase longevity in *C. elegans* and *Drosophila* [28], suggesting that up-regulation of Hsp26 by DR may be causally linked to lifespan extension. To validate the proteomic results, cell lysates were separated first by non-denaturing size-exclusion chromatography and subsequently by denaturing SDS–PAGE. As Hsp26 is known to form 24-subunit oligomers with a native molecular weight of ∼600 kDa, while having a small monomeric mass of 26 kDa [27], we reasoned that it should be possible to detect and distinguish the forms of Hsp26 using this approach.

**Figure 4.**
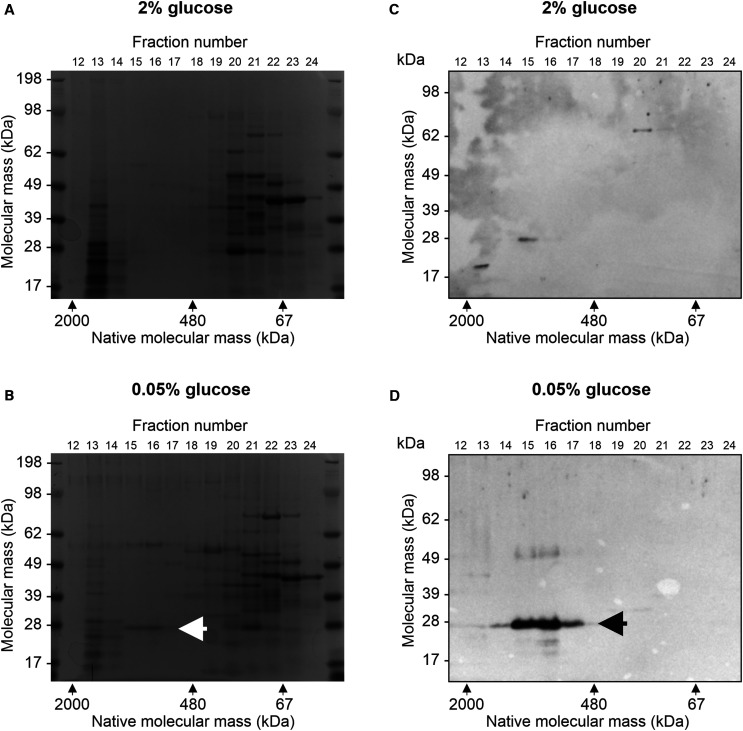
Hsp26 is strongly up-regulated by DR. BY4741 cells were grown in 2% or 0.05% glucose, lysed, diluted to a protein concentration of 1 mg/ml and injected onto a Superdex 200 size exclusion chromatography column. Eluted fractions were then separated by SDS–PAGE and either stained with Coomassie blue (**A** and **B**) or western blotted using a custom-made Hsp26 antibody (**C** and **D**). The arrow in **B** indicates a band of monomeric mass ∼28 kDa but native mass ∼600–700 kDa that was strongly induced by DR. A band of similar native and monomeric mass was detected by the Hsp26 antibody (indicated by the arrow in **D**), confirming its identity.

SDS–PAGE gels of lysates from standard and DR conditions exhibited a broadly similar protein expression pattern (Figure 4A,B). However, one band appeared in 0.05% glucose that was not detected in 2% glucose (arrow in Figure 4B). This band displayed a molecular mass of just under 28 kDa but migrated on size exclusion chromatography at ∼600–700 kDa, suggesting that it may be Hsp26. To verify this, western blotting was carried out using a custom-made antibody to the C-terminus of Hsp26 (Figure 4C,D). This revealed strong Hsp26 immunoreactivity of a similarly sized band under DR conditions but only minimal expression under standard conditions, confirming the quantitative proteomics findings. To determine how quickly Hsp26 protein levels decrease after the DR is relieved by a return to standard conditions, we performed western blotting on BY4741 wild type cells pre-grown to mid-log phase in 0.05% glucose before switching to 2% glucose. This revealed that Hsp26 protein levels decline rapidly following the switch — by ∼80% after 3 h and >90% after 5 h, averaged over three replicate experiments (Supplementary Figure S2). In contrast, no reduction in the control housekeeping protein alpha tubulin was observed. These reductions in Hsp26 are larger than can be explained by dilution with new cells formed during the incubation period (∼60% of total cells were newly formed at 3 h and 85% at 5 h), even if the newly formed cells expressed no Hsp26 protein. This suggests that switching from DR to standard 2% glucose conditions may trigger post-translational degradation of Hsp26 in pre-existing cells, in addition to repressing transcription of the *HSP26* gene.

**Figure 5.**
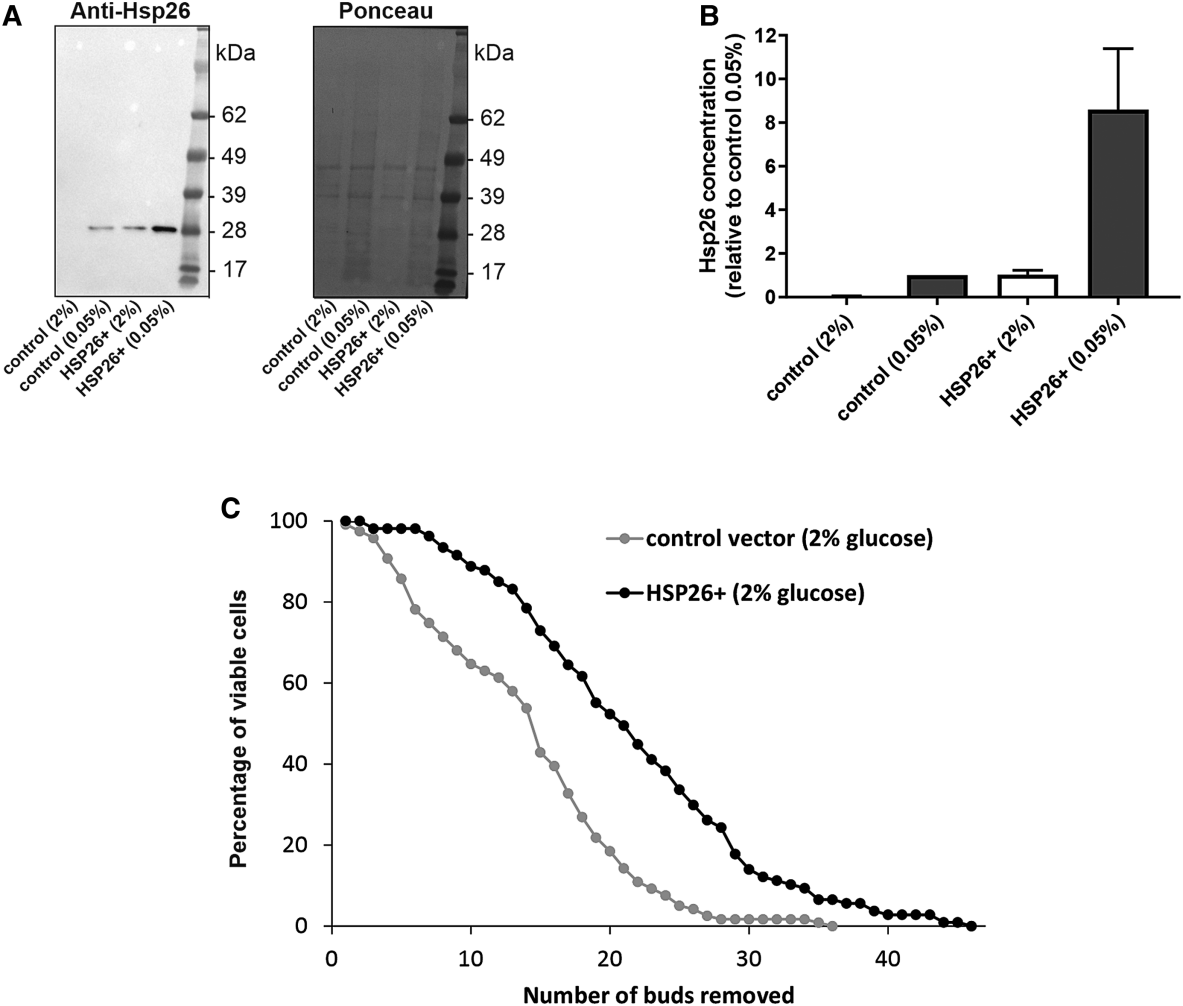
Hsp26 overexpression extends replicative lifespan. (**A**) BY4741 cells were transformed with a centromeric plasmid containing *HSP26* driven by the *ADH1* promoter *(*HSP26+) or empty vector (control). Transformed cells were then grown in -leu media containing 2% or 0.05% glucose, western blotted and probed with Hsp26 antibody (left panel) or stained for total protein with Ponceau S (right panel). (**B**) Quantification of Hsp26 expression normalised to total protein levels based on densitometry of three independent biological repeats. Data are shown relative to the mean Hsp26 concentration in the empty vector control cells in 0.05% glucose. (**C**) Replicative lifespan analysis of the Hsp26 overexpressing *(*HSP26+) and control vector strains described in **A** grown on -leu media containing 2% glucose. Data shown are pooled from four independent biological repeat experiments (control vector: *n* = 119 cells; HSP26+: *n* = 107 cells). Hsp26 overexpression resulted in a significant lifespan extension (*P* < 0.01).

To test whether the DR-induced increase in Hsp26 levels might contribute to replicative lifespan extension, we created a centromeric overexpression plasmid with *HSP26* controlled by the *ADH1* promoter. Western blotting revealed that cells containing this plasmid grown in -leu media containing 2% glucose expressed Hsp26 at similar levels to empty vector control cells in 0.05% glucose (Figure 5A,B), enabling us to test if elevating Hsp26 expression (to levels observed in DR) would increase longevity. Replicative lifespan analysis was performed on cells grown in standard 2% glucose conditions (in -leu media, to select for plasmid retention via the *LEU2* marker). Cells harbouring the Hsp26 overexpression plasmid exhibited significantly longer replicative lifespan than cells containing empty vector (Figure 5C), indicating that increased expression of Hsp26 alone can increase longevity. To determine whether Hsp26 was essential for DR-induced replicative lifespan extension, we replaced the *HSP26* open reading frame with a *nat*MX deletion cassette. There was no significant difference in replicative lifespan between wild type and *HSP26Δ* strains in either 2% or 0.05% glucose, and both strains exhibited significant lifespan extension in response to DR (Supplementary Figure S3). Taken together, these results indicate that increased expression of Hsp26 alone can extend lifespan, but that DR is able to increase longevity in the absence of Hsp26.

## Discussion

**Figure 6.**
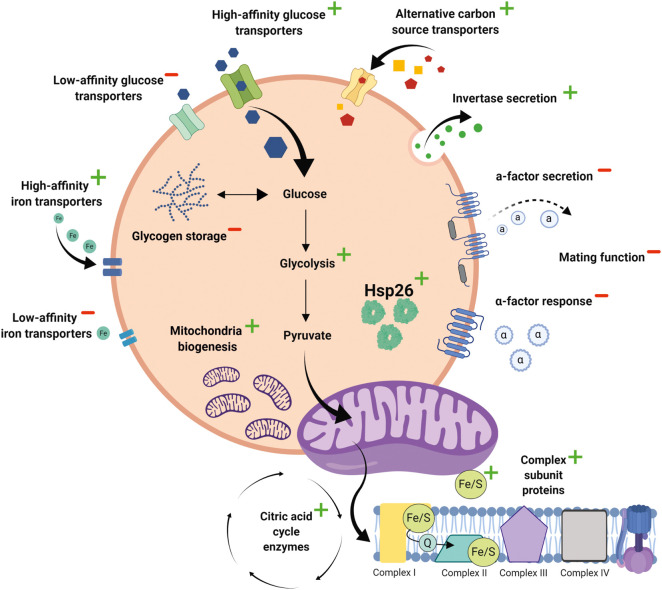
Physiological remodelling by DR. Cartoon illustrating the major DR-induced changes in cellular functions revealed by our proteomic analysis.

In this work, we employed label-free quantitative proteomics to characterise the effects of DR on global protein levels and validated these findings using orthogonal functional assays. These studies have revealed the coordinated changes in protein expression that underlie DR-induced physiological reprogramming. The picture that emerges is that yeast cells respond to DR by making better use of scarce energy sources, reducing mating activity and increasing replicative lifespan (Figure 6). In each of these cases, it is evident that multiple proteins and distinct mechanisms are employed. Cells respond to the low glucose environment in several ways. Expression of invertase (Suc2) was increased 13-fold, facilitating the conversion of extracellular sucrose into glucose and fructose; and the 73-fold elevation of glycogen debranching enzyme (Gdb1) would catalyse the breakdown of intracellular glycogen stores into glucose. In addition, DR increased the expression of high-affinity glucose transporters (15- and 20-fold increase in Hxt6 and Hxt7, respectively) and alternative carbon source transporters (26-fold increase in Jen1), whilst simultaneously down-regulating low affinity glucose transporters (17-fold decrease in Hxt3 expression). Taken together this suggests a coordinated response whereby cells respond to DR by more efficient scavenging of extracellular carbon sources and increased generation of intracellular glucose.

Our proteomic data indicate that DR also induces major changes to how intracellular glucose is utilised. For example, the first step in glucose metabolism is phosphorylation by either hexokinase 1 (Hxk1), hexokinase 2 (Hxk2) or glucokinase (Glk1), and all three proteins were significantly up-regulated by DR (20-fold, 2.5-fold and 6-fold increase, respectively). However, by far the clearest effect of DR was the switch in glucose metabolism from fermentation to mitochondrial respiration, as evidenced by the increased expression of many different mitochondrial proteins and by direct functional assays of mitochondrial respiration. Indeed, the majority of proteins up-regulated 3-fold or greater by DR were localised to mitochondria; and many other differentially expressed proteins had indirect links to respiration. For example, the largest DR-induced change in protein expression detected by LC–MS was of the high affinity iron transporter, FIT2 (100-fold increase), whereas the low affinity iron transporter, FET4, was down-regulated 10-fold. These changes likely reflect the need for cells to scavenge iron more efficiently in DR conditions to support the large increase in respiration, given that iron/sulfur clusters are assembled in mitochondria and are required for oxidative phosphorylation [29].

The DR-induced switch from fermentation to respiration we observed here was first documented by the Guarente laboratory, who reported a 2-fold increase in respiration in cells grown in 0.5% glucose compared with standard 2% glucose [13]. The higher 4-fold increase in respiration that we observed likely reflects the stronger effect of 0.05% glucose, which provokes a larger lifespan increase than 0.5% glucose [6]. It is well established that a similar switch from fermentation to respiration also occurs during the diauxic shift, resulting in an up-regulation of the gloxylate cycle and gluconeogenesis [30]. Indeed, we found that DR increased the expression of enzymes involved in the glyoxylate cycle (5-fold increases in Mdh1 (malate dehydrogenase) and Icl1 (isocitrate lyase)) and gluconeogenesis (11-fold increase in Pck1 (phosphoenolpyruvate carboxykinase)). Interestingly, it has been reported that overexpression of Mdh1 increases replicative lifespan [31]. Nevertheless, there are clear differences between DR and the diauxic shift. The vast majority of energy available to yeast cells in standard YPD media comes from the 2% glucose supplied; reducing this concentration 40-fold to 0.05% in DR therefore greatly limits the availability of caloric substrates. This contrasts with the situation in the classical diauxic shift, where after consumption of available glucose, an abundance of caloric substrates remain in the form of ethanol produced from the earlier fermentation of glucose. Another intervention that has been shown to increase respiration and up-regulation of mitochondrial gene expression is inhibition of TOR signalling [32]. Intriguingly, BY4741 *tor1* deletion mutants in 2% glucose have the same (extended) replicative lifespan as wild type cells in 0.05% glucose; and 0.05% glucose cannot further extend replicative lifespan in *tor1* mutants [33]. This indicates that the same form of DR in the same background strain that we used is TOR-dependent. As inhibition of TOR activity has been strongly implicated in DR-mediated longevity in various organisms [5], this may be of relevance beyond the yeast model.

Although there are no published proteomic data of actively growing cells in 0.05% glucose (mimicking the situation during replicative lifespan), there has been a previous microarray analysis of mRNA expression in cells growing in 0.5% glucose. That study identified only 124 differentially expressed genes at a threshold of 1.5-fold [13], compared with 782 proteins meeting this cut-off in our proteomic study. This may reflect the different glucose concentrations used, but may also reflect differences between indirect transcriptomic approaches versus direct proteomic analysis. Nevertheless, some differentially expressed genes/proteins related to metabolism and mitochondrial respiration were common to both datasets, including Fit2, Hxk1, Ald4, and mitochondrial ribosomal subunits [13]. A more recent study of yeast cells grown in a non-dividing state under extreme glucose-limiting conditions found that over 40% of the differentially expressed proteins were involved in mitochondrial and/or respiratory functions [34]. Evidently, increased respiration is a consistent feature of growth in low glucose. However, it is clearly not required for replicative lifespan extension by DR, as the longevity increase in 0.05% glucose is unaffected by mutations that abolish mitochondrial respiration [6].

One of the hallmarks of DR in metazoans, ranging from worms through flies to rodents, is reduced reproduction [35]. This is widely thought to represent an evolutionary strategy to prevent limited resources being invested in offspring with little chance of survival in a nutrient-poor environment. However, in the yeast replicative lifespan model studied here, individual mother cells actually produce more offspring under DR than under standard conditions and so would appear to be an exception to this general rule. However, we show here that DR actively inhibits mating of MATa cells with cells of the opposite mating type (MATα) by two distinct mechanisms. The first, down-regulating expression of the α-factor pheromone receptor, Ste2, has previously been shown to occur in cells grown in low glucose [36,37]. However, the second mechanism, reducing secretion of the a-factor pheromone via down-regulation of its transporter, Ste6, has not previously been reported. DR therefore inhibits mating both by releasing less pheromone to signal mating competence to other cells and by simultaneously decreasing the ability to respond to a pheromone released by cells of the opposite mating type. Hence, the universal effect of DR on reproduction can indeed be applied to yeast, provided that this is specific to sexual reproduction via mating, as opposed to asexual reproduction via mitosis. It is tempting to speculate that this may reflect a more selfish universal underlying evolutionary strategy in nutrient-poor environments, whereby mating is avoided to reduce the risk of unfavourable genetic recombination with other individuals.

A major finding of our work is that DR up-regulates Hsp26 expression and that overexpressing Hsp26 in standard conditions increases replicative lifespan. Intriguingly, Hsp26 is one of only five validated long-lived asymmetrically retained proteins (LARPs), which accumulate in yeast mother cells during replicative ageing [38]; and Hsp26 has also been shown to increase in expression during chronological yeast ageing [39]. Furthermore, recent proteomic analyses showed that Hsp26 is one of the most highly up-regulated proteins in response to sub-lethal heat shock [16], an environmental manipulation known to extend yeast replicative lifespan [40]. Hsp26 is widely accepted to function as a ‘holdase' molecular chaperone, which initially acts to prevent aggregation of client proteins and subsequently assists in the protein refolding stage [41]. However, its broad substrate specificity means that the key physiological client proteins remain unclear; and GFP-tagging studies show Hsp26 to be have a general cytoplasmic distribution, with no obvious localisation to subcellular compartments [21]. Nevertheless, given that most proteins up-regulated by DR are mitochondrial, it is possible that a proportion of Hsp26 may (transiently) localise to mitochondria in order to help maintain mitochondrial protein folding. Although we demonstrated that replicative lifespan can be extended simply by raising Hsp26 levels, DR was nevertheless still able to increase longevity in *hsp26* deletion mutants. This may be due to compensatory mechanisms that can substitute for Hsp26 function in its absence. Hsp26 is a member of the conserved sHSP family, which are ATP-independent ‘holdase' chaperones that help maintain cellular proteostasis by controlling protein misfolding and aggregation [42]. Hence, it may be that changes in the activity of Hsp42 and/or other elements of the proteostasis machinery can compensate for the loss of Hsp26 in *hsp26* deletion mutants. Given that impaired proteostasis is one of the hallmarks of ageing, it is unsurprising that sHSPs have been strongly linked to ageing and age-related diseases [28]. Indeed, overexpression of HSP-16 in *C. elegans* [43] and of Hsp22 in *Drosophila* [44] have previously been shown to increase lifespan, thus mirroring our findings with yeast Hsp26. Hence, sHSPs represent one of very few protein families whose expression/activity correlates with lifespan in yeast, flies and worms. This in turn suggests that sHSPs may be similarly important effector proteins of DR and longevity in higher organisms, including humans. Finally, the dataset of DR-regulated proteins we have provided here may help identify other conserved effectors of longevity and so may be a generally useful resource for the gerontology field.

## Data Availability

The mass spectrometry proteomics data have been deposited to the ProteomeXchange Consortium via the PRIDE [45] partner repository with the dataset identifier PXD025004 and 10.6019/PXD025004.

## Open Access Statement

Open access for this article was enabled by the participation of University of Liverpool in an all-inclusive *Read & Publish* pilot with Portland Press and the Biochemical Society under a transformative agreement with JISC.

## Abbreviations

DDA: data-dependent acquisition
DR: dietary restriction
LARPs: long-lived asymmetrically retained proteins
NDLB: non-denaturing lysis buffer
NMR: non-mitochondria respiration capacity
SD-leu: synthetic dropout media lacking leucine
TFA: trifluoroacetic acid
