# Supplementary material for: Proteomic analysis of dietary restriction in yeast reveals a role for Hsp26 in replicative lifespan extension
Source: Biochem J. 2021 Dec 16;478(24):4153–67. doi: 10.1042/BCJ20210432 (PMC8786290; doi:10.1042/BCJ20210432)
Supplement: Supplementary Figures S1-S3 [file BCJ-478-4153-s1.pdf]

Campion et al Supplementary information

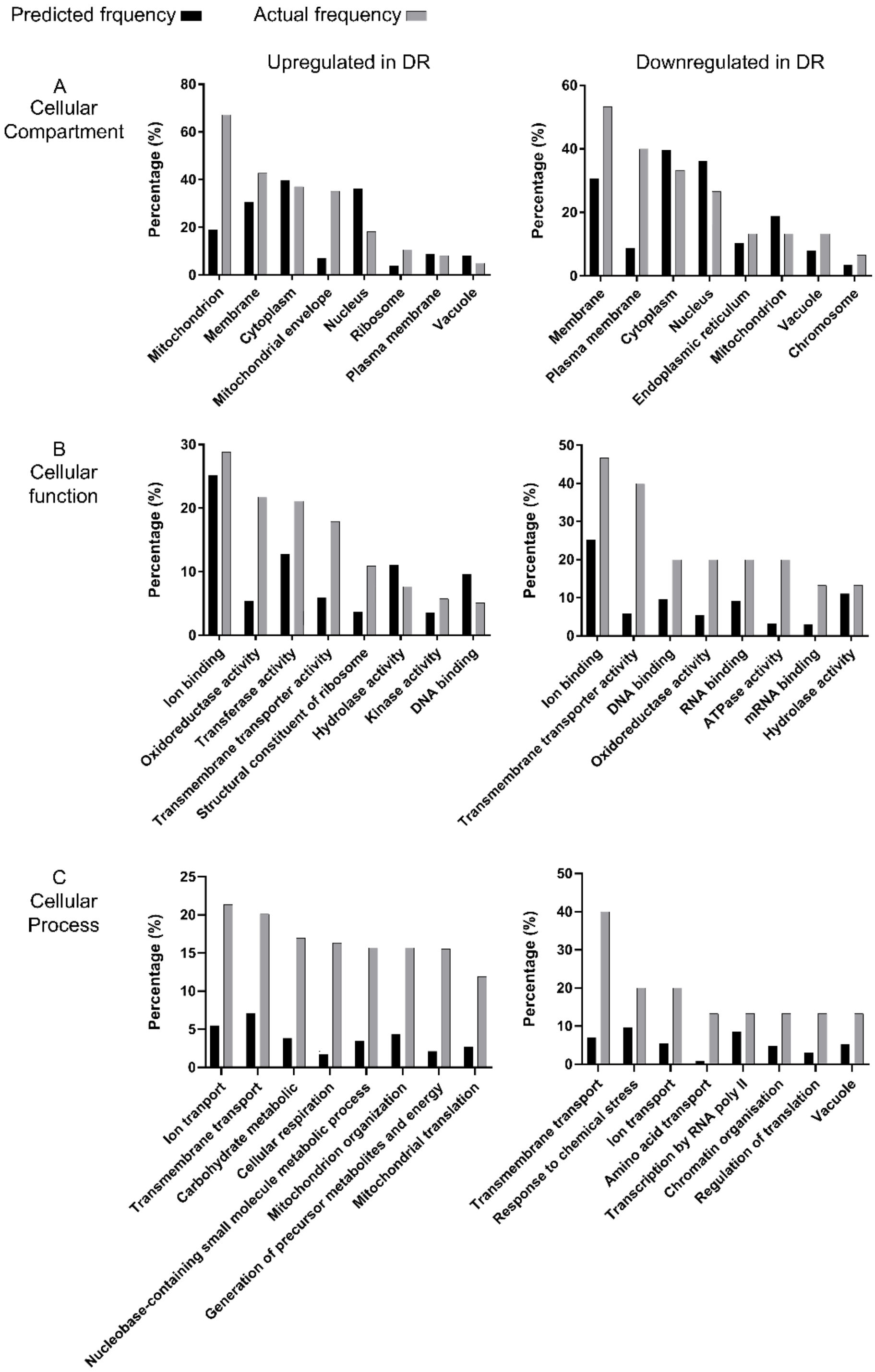

Supplementary Figure 1: GO term analysis of DR-regulated proteins.

Gene ontology (GO) analysis of the 183 significant protein with a 3-fold expression change during DR using GoSlimMapper. **A)** Cellular compartment analysis shows over-representation of mitochondrial proteins being upregulated, while membrane and plasma membrane proteins are downregulated during DR. **B)** Cellular function analysis shows over-representation of transporter and oxidoreductase enzymes proteins being upregulated, while opposing transporters proteins are downregulated during DR. **C)** Cellular processes analysis shows over-representation of transporters, metabolic, respiration and mitochondria processes being upregulated, while stress, chromatin organisation, translation and also transporter processes downregulated during DR.

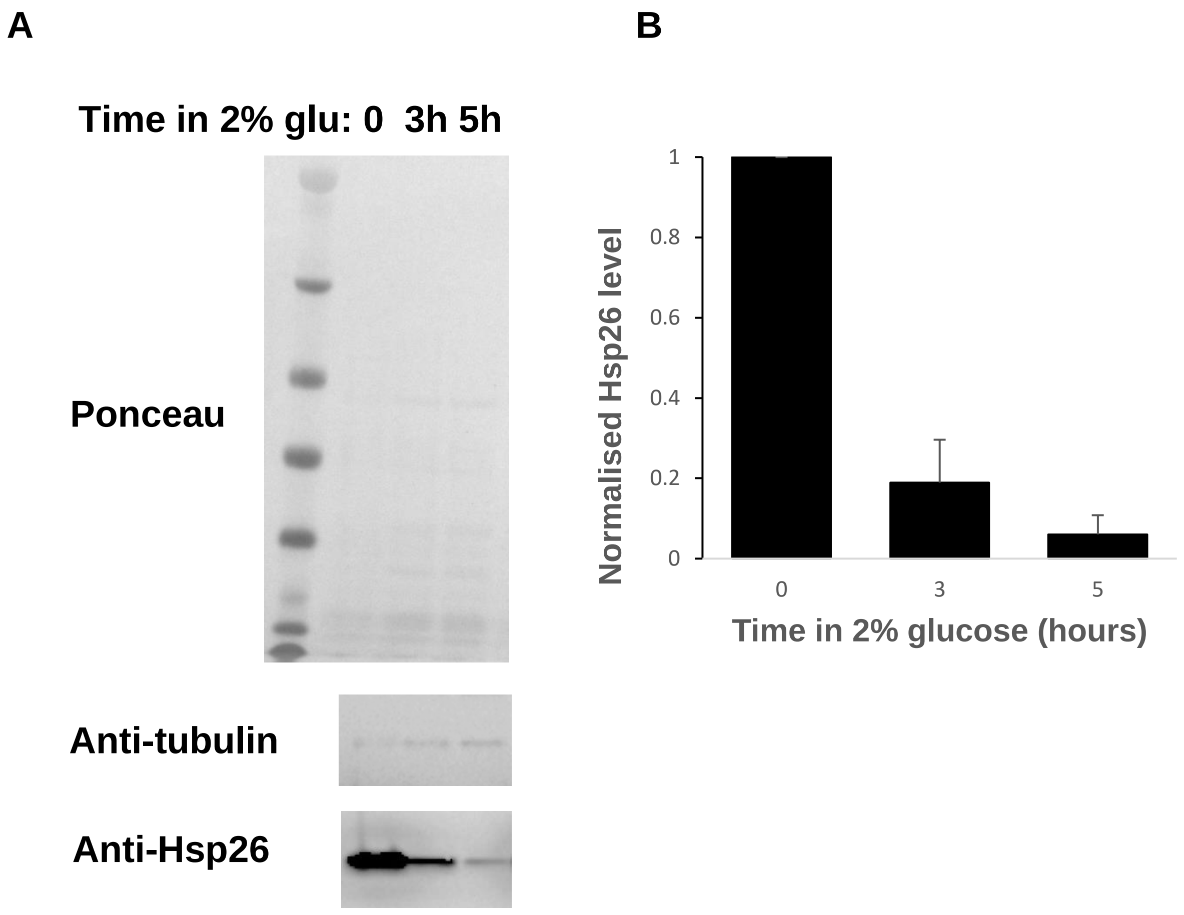

Supplementary Figure 2: Switching from dietary restriction to standard 2% glucose conditions triggers a rapid decline in Hsp26 levels.

BY4741 wild type cells were grown to mid-log phase in YPD media containing 0.05% glucose (time = 0) before switching to 2% glucose for 3 hours or 5 hours**. A)** Representative western blot showing total protein stain (Ponceau S), and immunoblotting using anti-alpha tubulin and anti-Hsp26 antibodies. **B)** densitometric quantification of Hsp26 levels normalised to total protein. Data shown are mean plus SEM (n=3 biological repeat experiments).

A

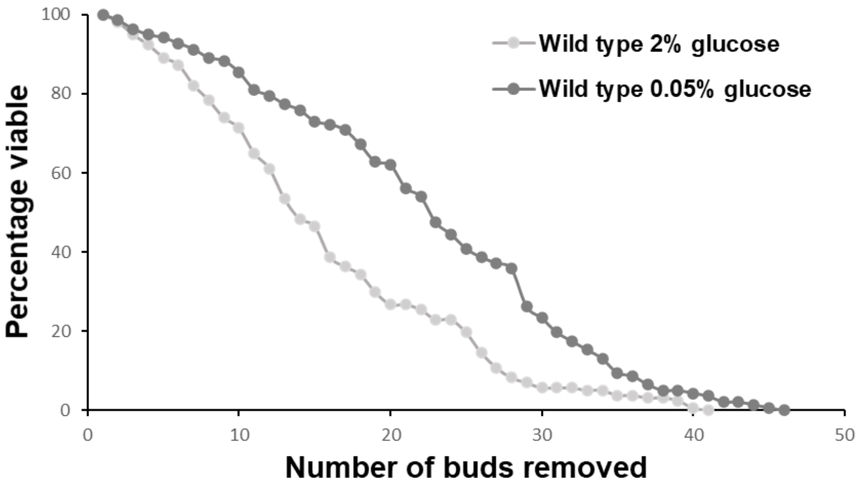

B

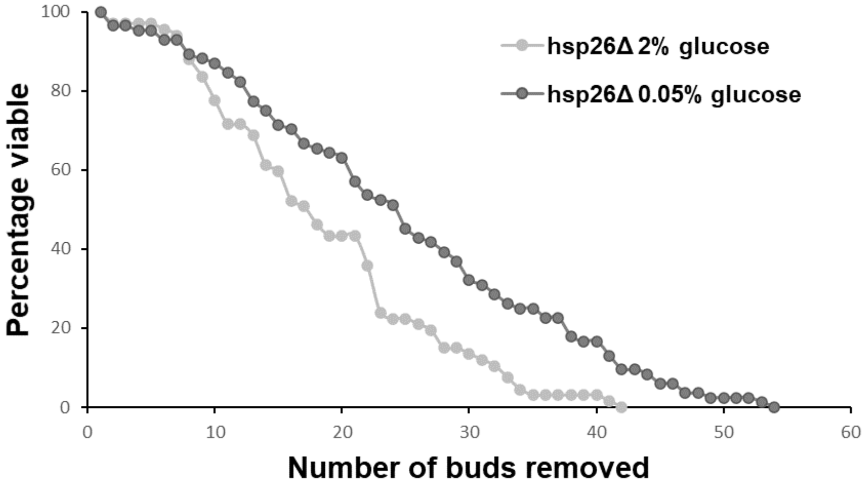

Supplementary Figure 3: Replicative lifespan analysis of wild type and *hsp26Δ* cells under standard and dietary restriction conditions.

Lifespan analysis was performed on BY4741 wild type (**A**) and *hsp26Δ* **(B)** cells grown on YPD media containing 2% or 0.05% glucose. Data shown are pooled from at least 3 independent biological repeat experiments (WT 2%, n=157 cells (4 experiments); WT 0.05%, n=137 cells (5 experiments); *HSP26Δ* 2%, n=67 cells (3 experiments); *HSP26Δ* 0.05%, n=84 (4 experiments)). DR resulted in a significant lifespan extension in both wild type and mutant strains (P< 0.01). There was no significant difference in lifespan between wild type and *HSP26Δ* strains in either condition.
